# Effects of autism on 30-year outcome of anorexia nervosa

**DOI:** 10.1186/s40337-021-00518-1

**Published:** 2022-01-09

**Authors:** Søren Nielsen, Sandra Rydberg Dobrescu, Lisa Dinkler, Carina Gillberg, Christopher Gillberg, Maria Råstam, Elisabet Wentz

**Affiliations:** 1 Psychiatric Research Unit, Psychiatry Region Zealand, Slagelse, Denmark; 2 Gillberg Neuropsychiatry Centre, Institute of Neuroscience and Physiology, University of Gothenburg, Gothenburg, Sweden; 3 Department of Child and Adolescent Psychiatry, University of Glasgow, Glasgow, UK; 4 Department of Clinical Sciences Lund, Child and Adolescent Psychiatry, Lund University, Lund, Sweden; 5 Department of Psychiatry and Neurochemistry, Institute of Neuroscience and Physiology, University of Gothenburg, Gothenburg, Sweden; 6 Högsbo Hospital, Lilla Kapplandsgatan 26, 42137 Västra Frölunda, Sweden

**Keywords:** Anorexia nervosa, Autism spectrum disorder, Long-term outcome, Psychosexual, Socioeconomic, Community-based, Controlled

## Abstract

**Background:**

Long-term consequences of comorbid autism spectrum disorder (ASD) in individuals with anorexia nervosa (AN) are inadequately investigated.

**Methods:**

In the 1980s, 51 adolescent-onset AN cases (AN group) and 51 matched controls (COMP group) were recruited from the community. They have been examined on five occasions. The four last assessments included the Morgan-Russell Outcome Assessment Schedule (MROAS) to assess eating disorder outcomes (weight, dieting, menstruation), and related problems including psychiatric, psychosexual and socioeconomic state. In the present study, at age 44, when 30 years had elapsed, MROAS data were compared with previous results. At age 16, 21, 24 and 32 years, all individuals had been assessed regarding ASD. At the 30-year follow-up, the impact of the ASD on the MROAS data was analysed.

**Results:**

In the AN group, all core anorectic symptoms (weight, dieting, menstruation) were on a par with the COMP group at the 30-year follow-up, but the positive outcomes were limited to those who had never had an ASD diagnosis. Psychiatric state was significantly worse in the AN group, particularly in the subgroup who had an ASD diagnosis assigned. The AN group—again particularly those with ASD—had a more negative attitude to sexual matters than the COMP group. The AN group had worse outcomes than the COMP group for ‘personal contacts’, ‘social contacts,’ and ‘employment record’ at the 30-year follow-up and the outcomes were worse the more often an ASD diagnosis had been assigned.

**Limitations:**

Rare data collection points throughout 30 years (only 5 assessments). ASD was assessed in the first four studies but was not assessed again at the 30-year follow-up.

**Conclusions:**

Mental health, psychosexual, and socioeconomic status were compromised up to 30 years after AN onset. Coexisting ASD contributed to the poor outcome. Core anorectic symptoms had “normalised” three decades after AN onset.

**Plain English summary:**

Some individuals with anorexia nervosa (AN) also suffer from autism. In this study we have investigated outcome of AN 30 years after the onset of AN and whether the presence of autism affects the outcome. Since the 1980s we have followed 51 individuals with teenage-onset AN and 51 healthy controls. They have been examined on five occasions, and an instrument that measures symptoms of AN (weight, dieting, body image), psychiatric symptoms, ability to work, and relationships with partner, family, and friends has been used to assess outcome. Autism was assessed in the first four studies. Symptoms of AN had normalised at 30-year follow-up, but only among those without autism. Psychiatric symptoms, ability to work, and relationships were issues that persisted after 30 years in the AN group, and those who had both autism and a history of AN had even more pronounced problems in these areas. The AN group had a more negative attitude to sexual matters than the control group, the outcome was worse the more often an autism diagnosis had been assigned.

**Conclusions:**

Mental health, psychosexual, and socioeconomic status are affected up to 30 years after AN onset, particularly among those with autism.

**Supplementary Information:**

The online version contains supplementary material available at 10.1186/s40337-021-00518-1.

## Background

Anorexia nervosa (AN) is characterised by starvation, underweight, fear of gaining weight, and a distorted body perception. It is one of the most severe psychiatric disorders that can affect a young person, due to a considerable risk of a chronic course and the highest mortality rate among all mental illnesses [1]. AN affects almost every vital organ, including the cerebral, cardiovascular, gastrointestinal, endocrinological, and musculoskeletal systems [2–4]. The increased mortality in AN is mainly caused by starvation, resulting in conditions such as treatment-refractory infections, cardiac arrest and kidney failure [5]. Comorbid alcohol use disorder and suicide are other well recognised causes of death in individuals with AN [1, 6].

Systematic data regarding very long-term psychosexual and socioeconomic consequences of AN are rare [7–10]. Ratnasuriya’s [7] and Löwe’s groups [8] followed up the psychosexual and socioeconomic status of inpatients with AN after 20 and 21 years, respectively. Fifty to sixty per cent of the former inpatients were living with a partner, and 50–71% were employed. In a German long-term outcome study, more than a thousand AN inpatients were followed for an average of ten years, including a subsample with 20-year outcome data [11]. At an average age of 34.5, only 27.4% of the women had become mothers compared with 57–74% of women of a similar age in the German general population [11]. Ratnasuriya et al. [7], using the Morgan Russell Assessment Schedule (MROAS) [12], observed that after 20 years, at mean age 41 years, one in three lived a very isolated life, and one in four had failed to emancipate from their family of origin. Non-eating disorder (ED) psychiatric morbidity at very long-term follow-up is dominated by affective and anxiety disorders [8, 13]. According to Dobrescu et al., 37.8% had a non-ED psychiatric diagnosis 30 years after the onset of AN [13]. Very long-term outcome studies pertaining to Health-Related Quality of Life (HRQoL) have shown poorer mental status in individuals with a history of AN [13], and poorer mental and physical HRQoL in individuals with chronic AN [14].

Autism spectrum disorder (ASD) has been reported to be overrepresented in chronic cases of AN [15]. In the Gothenburg AN study, we found that ASD in childhood or in young adulthood predicted a poor outcome at the 18-year follow-up of adolescent-onset AN [9]. The Gothenburg AN study has followed prospectively a group of adolescent-onset AN cases recruited from the community in 1985 [16]. MROAS has been used as an outcome measure at all follow-up examinations, and ASD diagnoses have been assessed since the original study in 1985 [17]. Nielsen et al. [10] analysed MROAS data including the effect of ASD over an 18-year follow-up period. Mental health, and psychosexual and socioeconomic state were poorer in the AN group, and the outcome was worse if an ASD was present. No other studies have previously followed prospectively a group of adolescent-onset AN cases for 30 years, and used the well-established outcome instrument MROAS at each follow-up examination. Furthermore, no one has hitherto investigated whether an ASD diagnosis will affect the outcome according to the MROAS 30 years after the onset of AN.

The aim of the present study was to investigate the course of the core anorexic traits, mental health, psychosexual and socioeconomic state in our AN group over a 30-year period and compare the data with the matched control group. We aimed specifically to analyse the effect of the diagnostic stability of ASD on the scales and subscales of the MROAS. Based on discouraging results pertaining to the psychosocial and socioeconomic outcome in previous long-term follow-up studies, we hypothesised that our AN group would still exhibit poorer results at the 30-year follow-up than their matched controls regarding mental health and psychosexual and socioeconomic state. We further hypothesised that a previously assigned ASD diagnosis would have a negative impact on MROAS, in general, and on the domains assessing mental health and psychosexual and socioeconomic state, in particular.

## Methods

### Participants

In the mid 1980’s an epidemiological study of the prevalence of adolescent-onset AN was conducted in Gothenburg, Sweden. All 4291 individuals born in 1970 and living in Gothenburg in 1985 were screened for previous and present AN. They all completed a questionnaire pertaining to ED symptoms and a researcher (Maria Råstam; MR) scrutinised all the growth charts. MR examined all individuals with a suspicion of an ED, resulting in 23 girls and two boys with AN. In-depth examination was declined by one of the girls leaving 22 girls and 2 boys to form the *population-based group*. Another group, the *population-screening group*, consisting of 27 individuals with adolescent-onset AN (26 girls, one boy) born in 1969, 1971–1975 and 1977, was formed after the cases had been reported to the researchers by the school health services. The *population-based group* and the *population-screening group* differed regarding ED treatment received, but virtually all other aspects were similar between the groups. The two groups were therefore merged to form the AN group consisting of 51 individuals; 48 girls and three boys (for further details see Råstam et al. 1989 and Råstam 1992) [16, 17]. All the AN cases fulfilled the criteria for AN according to the DSM-III-R [18] and the DSM-IV [19]. The mean age at onset of AN was 14.3 years. The school health services were also asked to select school-, age-, and sex matched comparison cases, without a history of ED. The comparison cases constituted the COMP group and included 51 individuals (48 girls and three boys) in line with the AN group format.

### Procedure

The 51 AN and 51 COMP cases were examined thoroughly for the first time at mean age 16 years (*AN Study 1*). The assessment included a collateral interview with the mother. The 102 cases (51 AN and 51 COMP) have thereafter been prospectively followed up at four occasions, at mean age 21, 24, 32, and 44 years [9, 13, 20, 21]. The follow-up periods were approximately 6 (*AN Study 2*), 10 (*AN Study 3*), 18 (*AN Study 4*), and 30 years (*AN Study 5*) after onset of AN, respectively. There was no attrition in *AN Study 2*, *AN Study 3,* and *AN Study 4*. In *AN Study 4*, there were 45 face-to-face interviews and five telephone interviews in the AN group. In one case, a mother was interviewed instead of the daughter, as the daughter was currently suffering from severe AN. In the COMP group, 48 individuals were interviewed in person, and three through telephone interviews. In the latest follow-up, *AN Study 5*, all but two women and two men in the AN group, and all individuals in the COMP group agreed to participate, corresponding to a dropout rate of 4% for the whole sample. In *AN Study 5* online video conferences or telephone interviews were conducted with eleven and nine of the individuals in the AN and COMP group, respectively. All the other participants were interviewed face-to-face.

ASD diagnoses were assigned in *AN Study 1*, *AN Study 2*, *AN Study 3*, and *AN Study 4*, each time by a new rater blinded to group status. The ASD diagnoses were based on structured interviews in all four studies. In addition, in *AN Study 2*, the Dewey social awareness test [22], and in *AN Study 4*, the self-report Autism-Spectrum Quotient questionnaire [23], were used. For details on the instruments used to assess symptoms of ASD see Nielsen et al. [10]. In *AN study 4* the individuals were grouped based on whether they had been assigned an ASD diagnosis in *AN Study 1*, *AN Study 2*, *AN Study 3*, and *AN Study 4* at one or several occasions. Individuals in the AN group with an ASD diagnosis at all four examinations were categorized as “ASD × 4” (n = 6), those who had been assigned an ASD diagnosis at least once and at most three times belonged to the “ASD × 1–3” category (n = 10), and the rest, those who had never fulfilled criteria for ASD, were classified as “never ASD” (n = 34). One woman in the AN group had experienced a severe head trauma in her early 20’s. She had previously been classified as having ASD, but in the present study she was excluded, since her psychiatric symptoms were not considered to have a childhood onset but acquired in early adult years. A woman in the COMP group was also removed from the study since her ASD diagnosis was considered secondary to a longstanding substance use disorder. The remaining 50 individuals in the COMP group were classified as “never ASD”. The thorough investigations of ASD traits in *AN Study 1* to *AN Study 4* resulted in no further diagnostic assessments of ASD in *AN Study 5*.

### Instruments

The semi-structured Mini International Neuropsychiatric Interview (MINI 6.0) [24] was used to interview all individuals regarding psychiatric disorders, both current disorders and disorders during the twelve-year period that had elapsed since the 18-year follow-up study (*AN Study 4*). The ED domain of the Structured Clinical Interview for DSM-IV (SCID-I) [25] was used as a complement to MINI 6.0, due to the additional questions regarding AN and binge-eating disorder. A checklist for DSM-5 EDs was included since the MINI and SCID-I interviews are based on DSM-IV criteria.

The Morgan-Russell outcome assessment schedule (MROAS) is a tool for estimating ED outcome, and was launched by Morgan & Russell in the 1970’s [26] and modified by Morgan & Hayward approximately a decade later [12]. The instrument comprises five scales (A to E), where scale A, D and E also include subscales. The questions focus on the previous six months. The scales/subscales are scored from 0 to 12 points. Scale A is entitled “Food intake”, with the subscales “A1. Dietary restriction” (0 corresponds to dietary restriction “at all times”, and 12 “nil” dietary restriction; 5 response categories), “A2. Worry about body weight or appearance” (0: worry “at all times”; 12: “nil” worry; 5 response categories), and “A3. Body weight” (0: “always much deviation sufficient to cause concern”; 12: “near average at all times”; 4 response categories). Scale B, named “Menstrual pattern (in previous 6 months)” (0: absent menstruations; 12: “regular and cyclic throughout”; 4 response categories). Scale C. “Mental state”, is based on what is observed at interview as well as reported abnormalities (0: “grossly abnormal and psychotic with delusions + hallucinations”; 12: “normal”; 4 response categories). Scale D. “Psychosexual state” consists of five subscales: “D1. Attitudes towards sexual matters” (0:”active dislike”; 12: “pleasurable”; 4 response categories), “D2. Professed aims in sexual relationships” (0: “wants to remain single”; 12: “definitely wants to marry and have children or has already done so”; 4 response categories), “D3. Overt sexual behaviour” (0: “avoids heterosexual contacts”; 12: “love affairs with pleasurable sexual relationship (may include married with children)”; 3 response categories), “D4. Attitude to menstruation (if it has returned)” (0: “active dislike”; 12: “pleased that it has returned”; 4 response categories), and “D5. Attitude to menstruation (if it has not returned)” (0: “pleased not returned”; 12: “regrets not returned”; 4 response categories). The D4 and D5 scales were not assessed in male participants. Scale E entitled “Socioeconomic state” is divided into five subscales: “E1. Relationship with nuclear family” (0: “very unsatisfactory”; 12: “satisfactory”; 4 response categories), “E2. Emancipation from family” (0: “many difficulties”; 12: “no difficulties”; 4 response categories), “E3. Personal contacts (apart from family and partner)” (0: “none”; 12: “many close and superficial friends”; 4 response categories), “E4. Social activities” (0: “nil outside family”; 12: “adequate group activities: mixes well outside family”; 4 response categories), and “E5. Employment record” (0: “no paid employment”; 12: “regular full time paid employment without absences”; 4 response categories). An average composite score of all scales (A + B + C + D + E) can be calculated. A separate scale, “Scale G. Self-progress rating”, is not included in the composite average score. It is rated by the patient and is scored from 0 to 3, where 0 corresponds to “worse” and 3 to “recovered”. In the present study we have concentrated on the responses from each individual on each scale and subscale of the MROAS. The MROAS has been widely used in research and in clinical practice, however, its psychometric properties indicate that it is best suited for AN compared with other EDs [12, 27]. The heteronormative perspective in Scale D has led to the omission of the scale in one study [28]. The measure Morgan Russell general outcome, categorised as good, intermediate or poor, based on weight and menstrual status, has been reported in a previous publication [13].

### Ethics

The Regional Ethical Review Board at the University of Gothenburg approved the study (398–14). All individuals participated voluntarily after giving written informed consent.

### Statistical analysis

This study is a longitudinal study, more specifically a repeated measures analysis. The individual scales and subscales of the MROAS have 3, 4 or 5 response categories, resulting in unbalanced multinomial tables with empty cells. The data structure does not permit the use of statistical methods relying on normality assumptions, consequently, we used exact non-parametric (permutation) statistical methods [29–32]. Computations were performed in the StatXact8 [33] software package on a PC platform. An ad hoc analysis, using Spearman’s rank order correlation coefficient [29], was performed to explore a possible correlation between subscale A2 (“Worry about body weight or appearance”) and subscale D1 (“Attitudes towards sexual matters”) in *AN Study 2*, *AN Study 3*, *AN Study 4*, and *AN Study 5*, based on a systematic review implying a relationship between body dissatisfaction and sexual dysfunction in EDs [34]. A more detailed description of the statistical procedures is presented as Additional file 1. All tests were two-tailed and conducted at a 5% significance level.

## Results

In the AN group, EDs had successively decreased between *AN Study 2* and *AN Study 4*; 20, 13, and 6 individuals had an ED in *AN Study 2, AN Study 3*, and *AN Study 4*, respectively. In *AN Study 5*, 9 individuals met criteria for an ED (AN: n = 3; Binge-eating disorder; BED: n = 1; Other Specified Feeding or Eating Disorder (OSFED): n = 5). In each follow up study, 3 individuals fulfilled criteria for AN. Only one individual fulfilled the criteria for an ED in the COMP group in *AN Study 5*, a case of OSFED, night eating syndrome. No one in the COMP group had met criteria for an ED in *AN Study 2*, *AN Study 3*, and *AN Study 4*. Between *AN Study 4* and *AN Study 5*, ten (21.3%) individuals in the AN group had had an ED relapse (AN: n = 5 (in one case, first BED and then AN); BED: n = 1 (not including the case that later developed AN); OSFED: n = 4). All the 102 individuals (51 AN and 51 COMP), including the 4 dropouts, were alive at 30-year follow-up. Two individuals in the AN group were in treatment for an ED in *AN Study 5*. Twenty-three percent had never received treatment for an ED.

### Overall test AN group versus COMP group

The generalised Cochran–Mantel–Haenszel test was significant for all but two subscales, justifying the detailed follow-up analyses. The two subscales that did not separate well between groups were D4 (“Attitude to menstruation (if it has returned)”, test statistic 2.6, exact *p* = 0.1) and E1 (“Relationship with nuclear family”, test statistic 2.79, exact *p* = 0.1). The generalised Cochran–Mantel–Haenszel test statistic for the remaining scales/subscales ranged from 8.0 to 56.4 (range of exact *p*: 0.003 to < 0.00001). Subscale D5 (“Attitude to menstruation (if it has not returned)”) could not be analysed as menses had returned in almost all individuals.

Table 1 provides an overview of all the scale and subscale results regarding comparisons between the AN and the COMP group and comparisons within the AN group based on the occurrence of coexistent ASD.

**Table 1:**
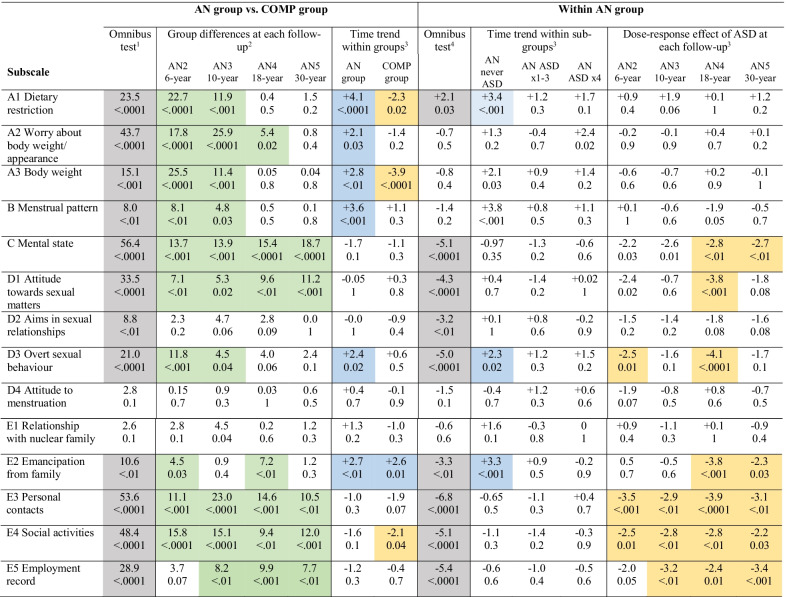
Overview of all results on the Morgan Russell outcome assessment schedule scale and subscale regarding comparisons between the AN and the COMP group and comparisons within the AN group based on the occurrence of coexistent ASD

Within each cell, the upper value represents the test statistic, and the lower value represents the exact *p* value. Significant omnibus tests are highlighted in grey. Follow-up tests are highlighted only if the omnibus test was significant. Positive trends are highlighted in blue, negative trends are highlighted in yellow, differences between the AN group and the COMP group are highlighted in green

AN: anorexia nervosa; COMP: comparison; ASD: autism spectrum disorder; AN 2: *AN Study 2*; six-year follow-up study (AN: n = 51; COMP: n = 51); AN 3: *AN Study 3*; 10-year follow-up study (AN: n = 51; COMP: n = 51); AN 4: *AN Study 4*; 18-year follow-up study (AN: n = 51; COMP: n = 51); AN 5: *AN Study 5*; 30-year follow-up study (AN: n = 47; COMP: n = 51). Year of data collection: *AN Study 2*: 1991–1992; *AN Study 3*: 1995–1996; *AN Study 4*: 2003–2004; *AN Study 5*: 2015–2016. ASD × 4: Individuals in the AN group with an ASD diagnosis at *AN Study 1* to *AN Study 4* (all four examinations) (n = 6); ASD × 1–3: those who had been assigned an ASD diagnosis at least once and at most three times (n = 10); never ASD: those who had never fulfilled criteria for ASD (n = 34)

Each statistical analysis was unique, involving a specific ‘research question’, and type I errors were therefore not taken into account

^1^Generalised Cochran–Mantel–Haenszel test for a singly ordered R × C table

^2^Kruskall-Wallis test for a single ordered R × C table

^3^Linear-by-Linear association test for a doubly ordered R × C table

^4^Generalised Cochran–Mantel–Haenszel test for a doubly ordered R × C table

### Core anorectic symptoms

Subscale A1—Dietary restriction, the distribution across response categories for this variable showed no inter-group difference in *AN Study 4* and *AN Study 5*. Significant inter-group differences were however observed in the first two follow-up studies [10], and were due to a significant improvement in the AN group, but limited to the subgroup ‘never ASD’ (*p* = 0.0008). Subscale A2—Worry about body weight or appearance, indicated large or evident differences between the AN and the COMP group in the previous follow-up studies, but the difference between the two groups was not significant in *AN Study 5*. Subscale A3—Body weight, exhibited a large difference between the AN and the COMP group in *AN Study 2* and *AN Study 3*, but no significant difference in *AN Study 4* and *AN Study 5*. This progress was due to a development towards a more normalised weight in the AN group, and in the COMP group a weight less often near average. The positive development in body weight in the AN group was limited to the group ‘never ASD’ (*p* = 0.03).

#### Scale B—Menstrual pattern

There were no between-group differences in *AN Study 5*. The normalised menstrual pattern in the AN group, was only due to the subgroup ‘never ASD’, which exhibited the positive development (*p* = 0.0001).

### Mental state

#### Scale C—Mental State

The between-group differences were highly significant in all four follow-up studies, with many low score responses in the AN group (Table 2). There was no change over time. A statistically significant, negative dose–response relationship was found in all follow-up studies; i.e., the more often an ASD had been assigned, the worse the outcome (Table 3).

**Table 2:** Morgan-Russell scale C (Mental state); comparisons between AN and COMP group at follow-up after 6, 10, 18 and 30 years

	Mental state								Singly ordered R × C Contingency Tables	
Follow-up	AN group				COMP group				Kruskal–Wallis test	Inference
	Response category				Response category				Test statistic	Exact *P *value
Carlo	0	4	8	12	0	4	8	12		
6-year	1	10	13	27	0	0	8	43	13.72	0.00015
10-year	1	10	14	26	1	2	4	44	13.91	0.00016
18-year	2	10	14	25	1	0	7	43	15.43	0.00006
30-year	2	13	15	17	1	2	8	40	18.71	0.00001

Doubly ordered R × C	Linear-by-linear association test		Stratified R × C Contingency Tables								
			Cochran–Mantel–Haenszel Test								
Inference	Test statistic −1.653	Test statistic −1.147	Test statistic 56.41								
	Exact *p* = 0.1	Exact *p* < 0.28	Exact *p* < 0.00001								

AN: anorexia nervosa; COMP: comparison; R × C: rows and columns; 6-year: *AN study 2* (6-year follow-up); 10-year: *AN study 3* (10-year follow-up); 18-year: *AN study 4* (18-year follow-up); 30-year: *AN study 5* (30-year follow-up)

Raw data are situated in the middle of the table. Results of the initial omnibus test, a stratified Cochran–Mantel–Haenszel test for a singly ordered R × C table, are placed in the lower right corner. Results from the analyses for group differences, Kruskall-Wallis test, at each follow-up are placed to the right of the data. Below the data the analyses for trend within each group (AN or COMP) are shown (Linear-by-Linear Association test)

**Table 3:** Morgan-Russell scale C (Mental state); dose–response analysis in the AN group

	Mental state																Analysis for time trend	
																	Doubly ordered R × C tables	
																	Linear-by-Linear Association Test	
Dose		AN Study 2			AN Study 3				AN Study 4				AN study 5				Test Statistic	exact p
	Response category				Response category				Response category				Response category					
	0	4	8	12	0	4	8	12	0	4	8	12	0	4	8	12		
Never ASD	0	4	12	18	0	5	9	20	0	6	8	20	0	7	9	14	−0.9656	0.35
ASD × 1–3	0	3	1	6	0	1	4	5	0	2	3	5	0	5	2	3	−1.326	0.2
ASD × 4	1	3	0	2	1	3	1	1	2	1	3	0	2	1	3	0	−0.6396	0.6

Dose–response analysis	Linear-by-linear association test				Stratified R × C contingency table													
Doubly ordered R × C tables					Cochran–Mantel–Haenszel test													
Test statistic	−2.165	−2.562	−2.787	−2.695	Test statistic −5.119													
Exact *p*	0.03	0.01	0.006	0.008	Exact *p* < 0.00001													

AN: anorexia nervosa; R × C: rows and columns; AN study 2: 6-year follow-up; AN study 3: 10-year follow-up; AN study 4: 18-year follow-up; AN study 5: 30-year follow-up; ASD × 4: Individuals in the AN group with an ASD diagnosis at *AN Study 1* to *AN Study 4* (all four examinations) (n = 6); ASD × 1–3: those who had been assigned an ASD diagnosis at least once and at most three times (n = 10); never ASD: those who had never fulfilled criteria for ASD (n = 34)

Raw data are situated in the middle of the table. Results of the initial omnibus test, a stratified Cochran–Mantel–Haenszel test for a doubly ordered R × C table, are placed in the lower right corner. Results of the analyses for time trend, the Linear-by-Linear Association test, are placed to the right of each level of ‘dose’. Below each follow-up study we have placed the results of the ‘dose–response’ analyses, using the Linear-by-Linear Associations test

### Psychosexual state

Subscale D1—Attitude towards sexual matters, showed a significant between-group difference in all four follow-up studies (*AN Study 2:* *p* = 0.006; *AN Study 3*: *p* = 0.02; *AN Study 4*: *p* = 0.002; *AN Study 5*: *p* = 0.0009). Subscale D2—Professed aims in sexual relationships, had no significant between-group differences in *AN Study 5*. Subscale D3—Overt sexual behaviour, exhibited significant between-group differences in *AN Study 2* (*p* = 0.0006) and *AN Study 3* (*p* = 0.04), but no difference in *AN Study 5*. Subscale D4—Attitude to menstruation, if it has returned, indicated that the two groups developed in a similar way, and no significant between-group differences were seen at any follow-up.

### Socioeconomic state

#### Subscale E1—Relationship with nuclear family

In *AN Study 2* and *AN Study 3* the relationships were reported unsatisfactory in the AN group. Differences between groups were no longer present in *AN Study 4* and *AN Study 5*. No significant dose–response relationships were found regarding ASD in any follow-up.

#### Subscale E2—Emancipation from family

Between-group differences were not significant in *AN Study 5* (*p* = 0.3). A significant improvement was only seen in the subgroup ‘never ASD’ (p = 0.0008). A significant negative dose–response relationship was visible in *AN Study* 5 (*p* = 0.025); the more often an ASD was assigned, the more difficult the emancipation from family (Table 4).

**Table 4:** Morgan-Russell subscale E2 (Emancipation from family); dose–response analysis in the AN group

Dose	Emancipation from family																Analysis for time trend	
																	Doubly ordered R × C tables	
																	Linear-by-linear association test	
	AN Study 2				AN Study 3				AN Study 4				AN Study 5				Test Statistic	Exact P
	Response category				Response category				Response category				Response category					
	0	4	8	12	0	4	8	12	0	4	8	12	0	4	8	12		
Never ASD	2	5	9	19	2	2	7	24	0	1	5	29	0	1	3	27	+ 3.269	0.0008
ASD × 1–3	0	2	0	8	0	1	1	8	0	1	2	7	0	0	1	9	+ 0.8723	0.5
ASD × 4	1	2	0	3	0	2	1	3	2	1	2	1	1	1	1	3	- 0.1564	0.9

Dose–Response analysis	Linear-by-linear association test				Stratified R × C contingency tables													
Doubly oredered R × C tables					Cochran–Mantel–Haenszel Test													
Test statistic	−0.517	−0.5369	−3.814	−2.314	Test statistic −3.301													
Exact P	0.7	0.6	0.0003	0.025	Exact *p* = 0.001													

AN: anorexia nervosa; R × C: rows and columns; AN study 2: 6-year follow-up; AN study 3: 10-year follow-up; AN study 4: 18-year follow-up; AN study 5: 30-year follow-up; ASD × 4: Individuals in the AN group with an ASD diagnosis at *AN Study 1* to *AN Study 4* (all four examinations) (n = 6); ASD × 1–3: those who had been assigned an ASD diagnosis at least once and at most three times (n = 10); never ASD: those who had never fulfilled criteria for ASD (n = 34)

Raw data are situated in the middle of the table. Results of the initial omnibus test, a stratified Cochran–Mantel–Haenszel test for a doubly ordered R × C table, are placed in the lower right corner. Results of the analyses for time trend, the Linear-by-Linear Association test, are placed to the right of each level of ‘dose’. Below each follow-up study we have placed the results of the ‘dose–response’ analyses, using the Linear-by-Linear Associations test

#### Subscale E3—Personal contacts

In all follow-up studies highly significant between-group differences were observed (see Table 5). A significant negative relationship was found in all four follow-up studies according to the detailed dose–response analysis (see Table 6).

**Table 5:** Morgan-Russell subscale E3; comparisons between AN and COMP group at follow-up after 6, 10, 18 and 30 years

	Personal contacts								Singly ordered R x C contingency tables	
	AN group				COMP group				Kruskal–Wallis Test	Inference
Follow-up	Response category				Response category				Test statistic	Exact *P value*
	0	4	8	12	0	4	8	12		
6-year	6	11	8	26	0	5	5	41	11.12	0.0009
10-year	4	20	7	20	0	6	1	44	23.02	< 0.00001
18-year	2	13	8	28	1	3	1	46	14.59	0.0001
30-year	4	23	3	17	2	12	1	36	10.47	0.001

Doubly ordered R x C tables	Linear-by-linear association test		Stratified R x C contingency tables							
			Cochran–Mantel–Haenszel Test							
Test statistic	−0.9814	−1.867	Test statistic 53.63							
	0.3	0.07	exact *p* < 0.00001							

AN: anorexia nervosa; COMP: comparison; R × C: rows and columns; 6-year: *AN study 2* (6-year follow-up); 10-year: *AN study 3* (10-year follow-up); 18-year: *AN study 4* (18-year follow-up); 30-year: *AN study 5* (30-year follow-up)

Raw data are situated in the middle of the table. Results of the initial omnibus test, a stratified Cochran–Mantel–Haenszel test for a singly ordered R × C table, are placed in the lower right corner. Results from the analyses for group differences, Kruskall-Wallis test, at each follow-up are placed to the right of the data. Below the data the analyses for trend within each group (AN or COMP) are shown (Linear-by-Linear Association test)

**Table 6:** Morgan-Russell subscale E3 (Personal contacts); dose–response analysis in the AN group

	Personal contacts																Analysis for time trend	
																	Doubly ordered R × C tables	
																	Linear-by-linear association test	
	AN Study 2				AN Study 3				AN Study 4				AN Study 5				Test statistic	Exact P
Dose	Response category				Response category				Response category				Response category					
	0	4	8	12	0	4	8	12	0	4	8	12	0	4	8	12		
Never ASD	2	5	5	22	2	9	6	17	0	6	4	24	0	13	2	15	−0.6536	0.5
ASD × 1–3	2	3	2	3	1	5	1	3	0	5	2	3	2	7	1	0	−1.117	0.3
ASD × 4	2	3	1	0	1	5	0	0	2	2	2	0	2	3	0	1	+0.4708	0.7

Dose–Response analysis	Linear-by-linear association test				Stratified R x C contingency tables													
Doubly oredered R x C tables					Cochran–Mantel–Haenszel test													
Test statistic	−3.462	−2.881	−3.851	−3.095	Test statistic—6.798													
Exact *P*	0.0004	0.004	0.00009	0.0015	exact *p* < 0.00001													

AN: anorexia nervosa; R × C: rows and columns; AN study 2: 6-year follow-up; AN study 3: 10-year follow-up; AN study 4: 18-year follow-up; AN study 5: 30-year follow-up; ASD × 4: Individuals in the AN group with an ASD diagnosis at *AN Study 1* to *AN Study 4* (all four examinations) (n = 6); ASD × 1–3: those who had been assigned an ASD diagnosis at least once and at most three times (n = 10); never ASD: those who had never fulfilled criteria for ASD (n = 34)

Raw data are situated in the middle of the table. Results of the initial omnibus test, a stratified Cochran–Mantel–Haenszel test for a doubly ordered R × C table, are placed in the lower right corner. Results of the analyses for time trend, the Linear-by-Linear Association test, are placed to the right of each level of ‘dose’. Below each follow-up study we have placed the results of the ‘dose–response’ analyses, using the Linear-by-Linear Associations test

#### Subscale E4—Social activities

The AN group had poor results in all follow-up studies, compared with the COMP group (*AN Study 5*: *p* = 0.0005). In all follow-up studies there was a significant negative dose–response relationship (*AN Study 5*: *p* = 0.03).

#### Subscale E5—Employment record

The between-group difference became significant in *AN Study 3* and in the subsequent follow-up studies (*AN Study 3*: *p* = 0.004; *AN Study 4*: *p* = 0.0015; *AN Study 5*: *p* = 0.005). The detailed dose–response analysis showed a clearly significant negative dose–response relationship from *AN Study 3* onwards (see Table 7).

**Table 7:** Morgan-Russell subscale E5 (Employment record); dose–response analysis in the AN group

	Employment record																Analysis for time trend	
	AN Study 2				AN Study 3				AN Study 4				AN Study 5				Doubly ordered R × C tables linear-by linear	
Dose	Response category				Response category				Response category				Response category				Test statistic	Exact *P*
	0	4	8	12	0	4	8	12	0	4	8	12	0	4	8	12		
Never ASD	3	3	5	24	4	1	3	27	8	2	6	19	0	3	11	17	−0.5697	0.6
ASD × 1–3	3	1	0	6	0	1	4	5	5	1	0	4	2	2	3	3	−0.9551	0.4
ASD × 4	3	0	0	3	4	1	0	1	4	0	1	1	4	0	0	2	−0.528	0.6

Dose–response analysis	Doubly ordered R x C tables				Stratified R × contingency													
	Linear-by-linear association test				Cochran–Mantel–Haenszel													
Test statistic	−2	−3.22	−2.437	−3.39	Test statistic—5.427													
Exact *p*	0.05	0.0015	0.014	0.0007	Exact *p* < 0.00001													

AN: anorexia nervosa; R × C: rows and columns; AN study 2: 6-year follow-up; AN study 3: 10-year follow-up; AN study 4: 18-year follow-up; AN study 5: 30-year follow-up; ASD × 4: Individuals in the AN group with an ASD diagnosis at *AN Study 1* to *AN Study 4* (all four examinations) (n = 6); ASD × 1–3: those who had been assigned an ASD diagnosis at least once and at most three times (n = 10); never ASD: those who had never fulfilled criteria for ASD (n = 34)

Raw data are situated in the middle of the table. Results of the initial omnibus test, a stratified Cochran–Mantel–Haenszel test for a doubly ordered R × C table, are placed in the lower right corner. Results of the analyses for time trend, the Linear-by-Linear Association test, are placed to the right of each level of ‘dose’. Below each follow-up study we have placed the results of the ‘dose–response’ analyses, using the Linear-by-Linear Associations test

Correlation between Subscale A2 (Worry about body weight or appearance) and Subscale D1 (Attitude towards sexual matters):

The AN group scored significantly worse than the COMP group on the scale Attitude towards sexual matters (D1) from mean age 21 to 44 years. According to a review by Castellini and colleagues [34], sexual dysfunction in women with AN is correlated with greater shape concerns. We therefore performed an ad hoc analysis to investigate whether there was a correlation between Scale A2 (Worry about body weight or appearance) and traits of sexual dysfunction (Scale D1). There were no correlations between Subscale A2 and Subscale D1, neither in the AN (*p* = 0.14), nor in the COMP group (*p* = 1.0) in *AN Study 5.* A correlation was found in the AN group in *AN Study 3* (*p* = 0.02) and *AN Study 4* (*p* = 0.036), and in the COMP group in *AN Study 3* (*p* = 0.007).

### Self-progress rating

#### Scale G—Self-progress rating

The probands’ self-evaluation of progress was much more positive than the interviewer’s evaluations. The test statistics from the generalised Cochran–Mantel–Haenszel test for doubly ordered R × C tables was + 0.1491, which gives an exact two-sided p value of 0.9. The test statistics from a linear-by-linear association test was + 1.54, which translates into an exact two-sided p value of 0.13. Accordingly, no statistically significant time trends or dose–response relationships were found.

## Discussion

In the present study, a 30-year follow-up of adolescent-onset AN, we hypothesised that Mental health, Psychosexual, and Socioeconomic state would still be poorer in the AN group than in the COMP group. In addition, we assumed that comorbid ASD, assessed in *AN Study 1* to *AN Study 4*, would result in poorer outcome evaluated by the MROAS, especially regarding the scales Mental state, Psychosexual, and Socioeconomic state, in the 30-year follow-up. Mental state (C), Attitudes towards sexual matters (D1), Personal contacts (E3), Social activities (E4), and Employment record (E5) all showed significantly worse outcomes in the AN group than in the COMP group at the 30-year follow-up. A dose–response relationship regarding ASD and outcome was observed for the Mental state scale (C), and the socioeconomic scales Personal contacts (E3), Social activities (E4) and Employment record (E5), indicating that the more often an ASD diagnosis had been assigned, the worse the outcome in those specific areas. The results pertaining to the ED-specific scales assessing dieting, menstruation, weight, and preoccupation with body shape and weight were no longer worse in the AN than in the COMP group. However, the improvement in the AN group was limited to those who had never fulfilled the criteria for an ASD.

The AN group reported more problems regarding Attitude towards sexual matters than the COMP group at all follow-up examinations. The results indicate that their libido is not on a par with that of their age-matched controls, despite the majority in the AN group reporting full ED symptom recovery in *AN Study 5* [13]. Comorbid ASD did not affect the participants’ attitudes towards sexual matters anymore. A significant correlation between Attitude towards sexual matters and shape concerns (Worry about body weight or appearance) was seen in *AN Study 3* in the COMP group, and in *AN Study 3* and *AN Study 4*, but not in *AN Study 5*, in the AN group. These findings are, however, difficult to interpret. Our analyses give, at most, limited support for the hypothesis presented by Castellini et al. [34], but the size of our groups limits the statistical power of our analyses. One may surmise that a relationship between excessive concern about body shape and sexual dysfunction is not exclusively an issue for women with an ED, but also for women without an ED.

The favourable results in the present study pertain to the core symptomatology of AN and other EDs, including weight, dieting, body shape and weight concerns, where no significant differences were found in the 30-year follow-up between the AN and the COMP group in these areas. We have previously published anthropometric data from the 30-year follow-up, which show that the mean BMI in the AN group is in accordance with the mean BMI in the COMP group [13]. However, the same publication reported that 19% (n = 9) in the AN group still suffered from an ED. Our prospective data have shown that Scale B (Menstrual pattern) and two out of three A scales (A1 Dietary restriction and A3 Body weight) had already “normalised” by *AN Study 4*, the 18-year follow-up, at mean age 32 years [9]. However, in terms of subscale A2 (Worry about body weight or appearance), it was not until the 30-year follow-up that the AN group scored in line with the COMP group. In a prospective follow-up study of female patients with AN, the participants were assessed after nine and 22 years [14]. The definition of recovery focused on not fulfilling any criteria for AN over the last year. Recovery from AN had occurred among 31.4% of the women at the nine-year follow-up, and among 62.8% after 22 years. The authors concluded that recovery from AN “continued over the long term” [14]. In terms of the course of core ED symptoms, we can report similar results and “Worry about body weight or appearance” may be one of the last symptoms to subside before recovery. In a follow-up study of female inpatients with AN twelve and 21 years after their first admission there was no significant improvement in ED symptoms between the two follow-up studies [8, 35]. This differs from the improved outcome of the core ED symptoms between the nine- and 22-year follow-up reported in the study by Eddy’s group [14], and a normalisation of core ED symptoms at 18- and 30-year follow-up in the present project.

The Mental health scale showed poorer results in the AN than in the COMP group at all follow-up examinations. The findings are partly due to the expected overrepresentation of EDs in the AN group, which is in line with a previous review of AN outcome studies, where 20% had developed chronic AN [36]. In *AN Study 3,* 27% still had an ED [21], in *AN Study 4* 12% had an ED [9], and in the present study, 19% met the criteria for an ED diagnosis [13]. However, over the years, the lower scores on the Mental health scale in the AN group might also be due to the overrepresentation of other psychiatric disorders, in particular, affective and anxiety disorders. The findings of psychiatric morbidity are in line with those of Löwe’s group [8], reporting on the outcome of AN 21 years after the first inpatient admission. In addition, Löwe et al. [8], and Halmi et al. [37] observed that alcohol use disorder was a relatively common problem in long-term follow-ups of AN; however, we could not replicate this finding since alcohol use disorder had been rare on all follow-up occasions [9, 13, 21]. In the 30-year follow-up, we found that the more often a diagnosis of ASD had been assigned, the worse the outcome was regarding the Mental health scale. This finding was also true with regard to *AN Study 2*, *AN Study 3* and *AN Study 4*, which probably reflects the pervasive impact an ASD has on an individual’s mental health.

Emancipation from family showed no improvement between the ten-year (*AN Study 3*) and the 30-year follow-up (*AN study 5*) in the subgroup of individuals in the AN group that had been assigned an ASD diagnosis on at least one occasion. In a Swedish register study, where female inpatients with adolescent-onset AN were followed up 9–14 years after admission, the women were significantly more often still living with their parents than age- and sex-matched individuals in the general population (9.0% versus 6.8%) [38]. One can surmise that ASD may have been overrepresented among the former AN inpatients in the register study, since ASD is more common among individuals with severe and enduring AN [15]. In any case, with an adult with AN who has not become independent of his or her parents, ASD should therefore be considered.

The socioeconomic subscales; i.e., Personal contacts, Social activities and Employment record, were still worse in the AN group compared with the COMP group at the 30-year follow-up. In the AN group, the presence of ASD contributed to the poorer outcome on these subscales. A study comparing social activities among women and men with ASD without intellectual disability found that women more often than men stated that they preferred their own company [39]. Our findings regarding less interest in taking part in social interplay are in line with the core symptomatology of ASD; however, a long-lasting ED together with another psychiatric morbidity probably also contribute to a limited social sphere.

‘Employment record’ had been worse in the AN than in the COMP group since *AN Study 3*. The more often an ASD diagnosis had been assigned, the worse the employment record during the last 20 years. Even poorer results in terms of employment record were reported in a 20-year follow-up study of AN patients from the 1990s, where half of the participants were unemployed [7]. Our discouraging Employment record results may have several explanations. Firstly, the information may reflect that some of those with ASD who had previously reported that they were engaged in university studies had later not been able to apply their skills in a workplace. Secondly, most individuals in the AN group no longer had small children and the individuals with ASD, who previously had been on full-time parental leave, now had difficulty finding suitable work, due to their social interaction impairment. Both examples indicate how individuals with previous or current AN might try to camouflage their poor psychosocial functioning from society’s expectations when it comes to coping with a full-time job.

### Strengths and limitations

This study has many strengths. We have followed a group of individuals with adolescent-onset AN for almost three decades. The mean age at AN onset was 14 years (30 years before the present study), and the first examination was performed at mean age 16 years. The study has a prospective case–control design; i.e., both the AN and the COMP group were given identical in-depth test batteries. All individuals were recruited from the community, and half the AN group constituted a total age cohort of adolescents attending 8^th^ grade at school in Gothenburg in 1985. No other AN sample has been followed up on so many occasions, and over such a long period of time. In our previous studies we have never experienced any dropout, and at the current assessment, 96% of the total sample participated, with four of the individuals in the AN group abstaining. Our research group’s early recognition of comorbid ASD in AN enabled us to assess ASD traits beginning already in the original study in 1985.

Some limitations should also be considered. Firstly, ASD was not assessed in the present study, and for this reason, we cannot know for certain whether the individuals with a previous ASD still fulfil the diagnostic criteria. We can, however, surmise that the diagnostic stability regarding the ‘ASD × 4’ group is fairly robust. Secondly, the sample consisting of 51 AN cases and 51 COMP cases may be considered relatively small. The modest sample size has, however, enabled us to conduct in-depth face-to face interviews at all examinations, and has made it possible to follow up every participant at each assessment, except four in the current study. The sample size limited the statistical power. Thirdly, this was not a treatment outcome study and therefore interventions were not systematically evaluated. Twenty-three per cent in the AN group reported that they had never received any treatment for an ED. However, the outcomes according to the Global Assessment of Functioning (GAF) [19] and the Morgan Russel averaged scale score did not differ between those who had ever received treatment for an ED and those who had not received treatment [13].

## Conclusions

Thirty years after the onset of AN, mental health, libido, social interaction and employment were still not on a par with the general population. The presence of ASD, diagnosed on one or several occasions, seemed to be one major reason why a minority of individuals with adolescent-onset AN never attained an acceptable level of psychosocial functioning. Among those with no history of ASD, the core ED symptomatology had normalised after three decades. In individuals who do not respond to evidence-based treatment of AN, screening procedures for ASD must be considered at an early stage.

## Supplementary Information

**Additional file 1:** Data structure and Statistical analyses.

## Data Availability

The datasets used and/or analysed during the current study are available from the corresponding author on reasonable request.

## Abbreviations

AN: Anorexia nervosa
ASD: Autism spectrum disorder
COMP: Comparison
ED: Eating disorder
HRQoL: Health-Related Quality of Life
MROAS: Morgan Russell Assessment Schedule
