# Supplementary material for: Effects of autism on 30-year outcome of anorexia nervosa
Source: J Eat Disord. 2022 Jan 9;10:4. doi: 10.1186/s40337-021-00518-1 (PMC8744255; doi:10.1186/s40337-021-00518-1)
Supplement: Supplementary file 1 — Additional file 1: Data structure and Statistical analyses. [file 40337_2021_518_MOESM1_ESM.docx]

Supplement S1 Data structure and Statistical analyses

Data structure

The individual scales and subscales of the Morgan-Russell outcome assessment schedule (MROAS) have 3, 4 or 5 response categories, making the resulting multinomial tables unbalanced with empty cells.

Statistical analysis

This study is a longitudinal study, more specifically a repeated measures analysis. The data structure does not permit the use of statistical methods, designed for ‘large’ samples relying on normality assumptions. Consequently, we used exact nonparametric (permutation) statistical methods (Agresti, 2002; Landis et al., 1978; Mantel & Byar, 1978; Siegel & Castellan Jr, 1988). Computations were performed in the StatXact8 software package on a PC platform (Cytel, 2007).

Firstly, analyses of ‘stratified singly ordered row by column (*r x c*) contingency tables’ were performed. There are four strata, one for each follow-up (*AN Study 2, AN Study 3, AN Study 4*, and *AN Study 5*)*.* There are two groups, the AN and the COMP group. A generalised Cochran-Mantel-Haenszel Test was used. *H*_0_: no association between the *r* rows (AN and COMP groups), and the *c* columns (response categories) in any of the *s* strata (*AN Study 2, AN Study 3, AN Study 4*, and *AN Study 5*).

Secondly, group differences at each point in time were analysed. The tables are ‘singly ordered *r x c* contingency tables’ (the rows (AN or COMP) are unordered, but the columns, the response categories, are ordered) and therefore the Kruskall-Wallis Test was used. *H*_0_: the multinomial probabilities across the response categories are identical in the two groups.

Thirdly, we performed the analysis for change over time. Each group is analysed separately. Data are arranged as ‘doubly ordered *r x c* contingency tables’, and thus the applicable statistical test is the Linear-by-Linear Association Test. The rows correspond to the different follow-up studies, and the columns are the responses. *H*_0_: identical multinomial probabilities at the four points in time; i.e., at *AN Study 2, AN Study 3, AN Study 4*, and *AN Study 5*. The selected statistical test is appropriate for detecting deviation from the null hypothesis *H*_0_ of the form *H*_1_ or *H´*_1_, or for detecting the two-sided alternative that either *H*_1_ or *H’*_1_ is true. Hypothesis *H*_1_ indicates that as one moves from one row to the next, the probability of the response falling in the next higher category rises. Hypothesis *H´*_1_ states the opposite. A large positive value of the test statistic rejects *H*_0_ in favour of *H*_1_ (i.e., a ‘better’ outcome), while a large negative value rejects *H*_0_ in favour of *H´*_1_ (i.e., a ‘worse’ outcome). The results of the statistical tests are located below each group at the bottom of each table.

Fourthly, data of the AN group in a dose-response model in relation to ASD were analysed. ‘Dose’ corresponds to ASD diagnoses, ordered hierarchically into three groups: ‘never ASD’, ‘ASD x 1-3’, ‘ASD x 4’. ‘Response’ is the distribution across the existing response categories. The principal investigators hypothesised, before any analyses were performed, that mental state, psychosexual state and socioeconomic state were likely to be influenced by the ASD diagnostic stability, and therefore data were organised in ‘doubly ordered *r x c* contingency tables’, and accordingly the appropriate test was a ‘Linear-by-Linear Association Test’. These analyses did not include the COMP group.

The analyses of the G scale (Self-progress rating) encompassed only the AN group for obvious reasons (Agresti, 2002; Landis et al., 1978).

An ad hoc analysis was performed regarding a possible correlation between subscale A2 (Worry about body weight or appearance) and subscale D1 (Attitude towards sexual matters) in *AN Study 2*, *AN Study 3*, *AN Study 4*, and *AN Study 5*, based on a systematic review implying a relationship between body dissatisfaction and sexual dysfunction in eating disorders (Castellini et al., 2016). Since subscale A2 has five response categories (0, 3, 6, 9, 12), and D1 has four categories (0, 4, 8, 12), all categories but 0 and 12 were ‘discordant’, and therefore the Spearman’s rank order correlation coefficient was used.

*Power analyses*

Exact power of linear rank test for comparing two ordered multinomial populations (StatXact8; Cytel, 2007) indicated that the statistical analyses were sufficiently powered.

Output from the power analysis for 5, 4 and 3 response categories, respectively, is presented below.

*Exact power of linear rank tests for comparing two ordered multinomial populations*

Type I error (Alpha) = 0.05

Number of populations, that is, response categories, (K) = 5

Sample Size in each population:

Pop 1 (m1) = 51

Pop 2 (m2) = 51

Pop 2 probabilities: Adjacent categories model (Slope = 1)

Pop 1 (Pi1): 0.2 0.2 0.2 0.2 0.2

Pop 2 (Pi2): 0.01166 0.03168 0.08613 0.2341 0.6364

Scores: 1 2 3 4 5

Compute: Two sided

Asymptotic Power = > 99.9%%

Exact Power = > 99.9%%

Two Ordered Multinomials: Power

*Exact power of linear rank tests for comparing two ordered multinomial populations*

Type I error (Alpha) = 0.05

Number of populations, that is, response categories,(K) = 4

Sample Size in each population:

Pop 1 (m1) = 51

Pop 2 (m2) = 51

Pop 2 probabilities: Adjacent categories model (Slope = 1)

Pop 1 (Pi1): 0.25 0.25 0.25 0.25

Pop 2 (Pi2): 0.03206 0.08714 0.2369 0.6439

Scores: 1 2 3 4

Compute: Two sided

Asymptotic Power = 99.93%

Exact Power = 99.84%

Two Ordered Multinomials: Power

*Exact power of linear rank tests for comparing two ordered multinomial populations*

Type I error (Alpha) = 0.05

Number of populations, that is, response categories,(K) = 3

Sample Size in each population:

Pop 1 (m1) = 51

Pop 2 (m2) = 51

Pop 2 probabilities: Adjacent categories model (Slope = 1)

Pop 1 (Pi1): 0.3333 0.3333 0.3333

Pop 2 (Pi2): 0.09003 0.2447 0.6652

Scores: 1 2 3

Compute: Two sided

Asymptotic Power = 97.08%

Exact Power = 95.89%

**References**

Agresti, A., 2002. Categorial Data Analysis, 2.ed. John Wiley & Sons, New York.

Castellini G, Lelli L, Ricca V, Maggi M. Sexuality in eating disorders patients: etiological factors, sexual dysfunction and identity issues. A systematic review. . Horm Mol Biol Clin Investig. 2016;25(2):71-90.

Cytel, I., 2007. StatXact8: Statistical Software for Exact Nonparametric Inference, User Manual. Cytel Software Corporation, Cambridge, MA, U.S.A.

Landis, J., Heyman, E., Koch, G., 1978. Average Partial Association in Three-Way Contingency Tables: A Review and Discussion of Alternative Tests. Int. Stat. Rev. 46, 237–254. https://doi.org/10.2307/1402373

Mantel, N., Byar, D., 1978. Marginal homogeneity, symmetry and independence. Commun. Stat. - Theory Methods 7, 953–976. https://doi.org/10.1080/03610927808827685

Siegel, S., Castellan Jr., N.J., 1988. Nonparametric statistics for the behavioral sciences, 2nd ed., McGraw-Hill, New York.
